# Expression patterns of the poplar *NF-Y* gene family in response to *Alternaria alternata* and hormone treatment and the role of *PdbNF-YA11* in disease resistance

**DOI:** 10.3389/fbioe.2022.956271

**Published:** 2022-09-16

**Authors:** Ying Huang, Huijun Ma, Xiaodong Wang, Tianxiang Cui, Gang Han, Yu Zhang, Chao Wang

**Affiliations:** State Key Laboratory of Tree Genetics and Breeding, Northeast Forestry University, Harbin, China

**Keywords:** poplar, NF-Y transcription factor, expression pattern, JA signal, disease resistance

## Abstract

Plant nuclear factor-Y (NF-Y) transcription factors (TFs) are key regulators of growth and stress resistance. However, the role of NF-Y TFs in poplar in response to biotic stress is still unclear. In this study, we cloned 26 *PdbNF-Y* encoding genes in the hybrid poplar *P. davidiana* × *P. bollena*, including 12 *PdbNF-YA*s, six *PdbNF-YB*s, and eight *PdbNF-YC*s. Their physical and chemical parameters, conserved domains, and phylogeny were subsequently analyzed. The protein–protein interaction (PPI) network showed that the three PdbNF-Y subunits may interact with NF-Y proteins belonging to two other subfamilies and other TFs. Tissue expression analysis revealed that *PdbNF-Y*s exhibited three distinct expression patterns in three tissues. Cis-elements related to stress-responsiveness were found in the promoters of *PdbNF-Y*s, and most *PdbNF-Y*s were shown to be differentially expressed under *Alternaria alternata* and hormone treatments. Compared with the *PdbNF-YB* and *PdbNF-YC* subfamilies, more *PdbNF-YA*s were significantly induced under the two treatments. Moreover, loss- and gain-of-function analyses showed that *PdbNF-YA11* plays a positive role in poplar resistance to *A. alternata*. Additionally, RT‒qPCR analyses showed that overexpression and silencing *PdbNF-YA11* altered the transcript levels of JA-related genes, including *LOX*, *AOS*, *AOC*, *COI*, *JAZ*, *ORCA*, and *MYC*, suggesting that *PdbNF-YA11*-mediated disease resistance is related to activation of the JA pathway. Our findings will contribute to functional analysis of *NF-Y* genes in woody plants, especially their roles in response to biotic stress.

## Introduction

To eliminate pathogens, plants have developed a complex defense system, of which phytohormones act as signals to trigger and mediate a diverse array of defense responses. Studies have shown that jasmonic acid (JA) triggers plant resistance by regulating the accumulation of ROS, callose, lignin and the expression of defense-related genes (Behr et al., 2018; Block et al., 2018; Moosa et al., 2019; Varsani et al., 2019). Salicylic acid (SA) is known to play a crucial role in systemic-acquired resistance (SAR) and plant innate immunity (Peng et al., 2021). Recently, transcription factors (TFs) were shown to be involved in plant disease resistance by regulating the hormone signaling pathway. For example, MYC2 and its downstream MYC2-targeted TFs (MTFs) form a transcription module that may directly regulate the JA-induced transcription of late defense genes during tomato resistance to *B. cinerea* (Du et al., 2017). *PeTGA1* from *Populus euphratica* was shown to play a key role in plant immunity by positively regulating both the SA biosynthesis pathway and SA signaling during *Colletotrichum gloeosporioides* infection (Yang et al., 2022). WRKY transcription factors, including AtWRKY75, AtWRKY28, WRKY50, WRKY51, WRKY70, WRKY62, WRKY3, WRKY4, WRKY33, WRKY18, WRKY40, WRKY48, WRKY60, and WRKY41, in *Arabidopsis* (Wang et al., 2006; Zheng et al., 2006; Knoth et al., 2007; Lai et al., 2008; Pandey and Somssich 2009; Chen et al., 2013; Chen et al., 2020), CaWRKY27 in *Capsicum annum* (Dang et al., 2014), and PtrWRKY89 and PtrWRKY73 in poplar (Duan et al., 2015; Jiang et al., 2016) were shown to play roles in disease resistance by regulating the SA- and JA/ET-mediated signaling pathways. However, compared with studies on other TFs, little is known about the roles of nuclear factor Y (NF-Y) in plant disease resistance.

Nuclear factor Y (NF-Y), also called heme activator proteins (HAPs) or CCAAT-binding factors (CBFs), is widespread and evolutionarily conserved in plants, fungi, and animals (Kim et al., 1996). Unlike the other transcription factors (TFs), NF-Y is a heterotrimeric complex consisting of three distinct subunits NF-YA (HAP2/CBF-B), NF-YB (HAP3/CBF-A), and NF-YC (HAP5/CBF-C). The subfamilies (NF-YA, NF-YB, and NF-YC) are characterized by their conserved domains and sequence lengths. Generally, NF-YA sequences with a core region composed of 53 amino acids that contains two helices (α1 and α2) are longer than those of NF-YB and NF-YC (Petroni et al., 2012; Laloum et al., 2013). Of the two helices, α1 is required for subunit interactions in the N-terminal side and α2 is located in the C-terminal for sequence specificity to bind DNA at the *CCAAT* boxes. NF-YB and NF-YC, which contain three α-helices (α1, α2, and α3) and another α-helix domain in the C-terminus (αC), share histone-fold domains (HFDs) with the core histones H2B and H2A, respectively (Romier et al., 2003; Hackenberg et al., 2012). When NF-Y regulates downstream target genes, NF-YB and NF-YC form a dimer in the cytoplasm first, and then this dimer is transferred into the nucleus, where it interacts with NF-YA to complete the assembly of the heterotrimeric complex, binding to the *CCAAT* box and finally regulating the expression of downstream target genes (Petroni et al., 2012; Laloum et al., 2013).

In plants, NF-Y has been validated to be involved in multiple physiological and developmental processes, such as embryogenesis, seed germination, flowering time, root growth, and fruit ripening. In *Arabidopsis thaliana*, *AtNF-YC3*, *-4*, and *-9* have been proven to control flowering time (Kumimoto et al., 2010); *AtNF-YB2* was shown to positively regulate primary root elongation (Ballif et al., 2011); *NF-YB9* has been shown to be a pivotal regulator in embryogenesis (Siefers et al., 2009); and NF-YCs were shown to integrate the GA and ABA signaling pathways by interacting with RGL2 during seed germination (Liu et al., 2016). In *Chrysanthemum*, *CmNFYB8* was shown to be involved in flowering time by directly regulating the expression of *cmo*-MIR156 (Wei et al., 2017). In *Triticum monococcum*, the *NF-Y* regulates flowering initiation by integrating vernalization and photoperiod seasonal signals (Li et al., 2011). Overexpression of *PtoNF-YC6* and *PtoNF-YC8* from *Populus tomentosa* showed early flowering (Li et al., 2020), and *PdNF-YB21* in poplar was shown to positively regulate root growth (Han et al., 2021). Moreover, plant NF-Ys are also involved in abiotic stress responses, especially salt or drought stress. For example, members of the *NF-YA* subfamily were proven to participate in drought resistance, such as *AtNF-YA2*, *AtNF-YA3*, and *AtNF-YA5* in *Arabidopsis* (Leyva-González et al., 2012). *StNF-YC9* played an important role in drought tolerance by increasing photosynthesis rate, antioxidant enzyme activity, and proline accumulation coupled to lowered malondialdehyde accumulation in potato (Li et al., 2021). Moreover, overexpression of *OsNF-YA7* in transgenic rice plants increased drought tolerance by modulating gene regulation in an ABA-independent manner (Dong-Keun et al., 2015). Heterologous expression of both *CdtNF-YC1* from *Bermuda grass* and *TaNF-YA10–1* from wheat resulted in enhanced drought and salinity tolerance (Chen et al., 2015; Ma et al., 2015). The accumulation of *GmNFYA* in soybean may lead to derepression and maintenance of histone acetylation for activation of salt-responsive genes, which ultimately confer salt tolerance (Lu et al., 2021). In addition, several studies have also shown that *NF-Y*s play roles in plant interactions with pathogenic microorganisms. For example, the deregulation of *NF-YA* enhanced resistance to *Ralstonia solanacearum* in *Arabidopsis* (Sorin et al., 2014), and *MtNF-YA1* appears to be a repressor of responses triggered in *Medicago truncatula* by *Aphanomyces euteiches* invasion (Rey et al., 2016). Both *ZmNF-YA3* and *ZmNF-YA8* in maize were upregulated after infection by the pathogens *F. moniliforme*, *S. reiliana*, and *C. graminicola* (Zhang et al., 2016). Moreover, the overexpression of *OsHAP2E* conferred resistance to pathogens of transgenic rice (Alam et al., 2015), and overexpression of *GmNF-YC4*-2 in soybean increased broad disease resistance for bacterial, viral, and fungal infections in the field (O’Conner et al., 2021). In cassava, the NF-Y transcription factor complex (MeNF-YA1/3, MeNF-YB11/16, and MeNF-YC11/12) was shown to play a role in the transcriptional activation of *MePR* promoters, contributing to the plant defense response (He et al., 2020).

*Populus* spp. are important landscape tree species around the world, that also have great economic and ecological value. However, abiotic stresses, such as cold injury and salinity, and biotic stresses, such as *Alternaria alternata*, *Melampsora larici-populina*, and *Dothiorella gregaria*, could cause damage to poplars and reduce their production. However, to date, few studies of the NF-Y family concerning biotic stress resistance have been performed in *Populus*. As such, to obtain a better understanding of the *NF-Y* family and boost future studies of this family with regard to resistance to biotic stress in *Populus*, we identified and characterized the *NF-Y* gene family in *P. davidiana* × *P. bollena*.

In this study, 26 *NF-Y* candidate genes were cloned and subjected to comprehensive analysis, including phylogenetic analysis, conserved motifs, promoter analysis, protein–protein interaction (PPI) network, and expression pattern analysis under biotic treatments. To further study this possibility, the function of *PdbNF-YA11* was characterized in transgenic poplar. The results of *PdbNF-Y* analysis provided a basis for further studies on the functions of the *PdbNF-Y* family in poplar disease resistance.

## Materials and methods

### Identification and bioinformatics analysis of *PdbNF-Y*s from the transcriptomes of *P. davidiana* × *P. bollena*

Amino acid sequences of *Populus trichocarpa* PtNF-Ys were obtained from PlantTFDB (https://planttfdb.cbi.pku.edu.cn/blast.php). The sequences were used for a local BLAST search of *P. davidiana* × *P. bollena* transcriptomes. Additionally, the hidden Markov model (HMM) profile (PF02045 and PF00808) of the NF-Y conserved domain was used to search the protein sequences from poplar transcriptomes. The results of the two methods were merged to obtain candidate *PdbNF-Y* members and then submitted to the CCD (https://www.ncbi.nlm.nih.gov/cdd/) and SMARAT (https://smart.embl-heidelberg.de/) programs to verify the complete NF-Y domains.

Physical and chemical parameters of the PdbNF-Y proteins were predicted using the ExPASy-ProtParam tool (https://web.expasy.org/protparam/). Multiple sequence alignment of PdbNF-Ys conserved domain sequences and the formation of sequence logos by Clustal Omega (https://www.ebi.ac.uk/Tools/msa/clustalo/) and WebLogo program (https://weblogo.berkeley.edu/logo.cgi) were performed (Crooks et al., 2004). A neighbor-joining (NJ) tree was constructed by MEGA 5.0 software (https://www.megasoftware.net/index.php) with 1000 bootstrap replicates. The MEME program (https://meme-suite.org/tools/meme) was used to detect the conserved motifs in PdbNF-Y proteins with the following parameters: the maximum number of motifs was 10 and the length range was 6–100 amino acids. Exon–intron structure visualization was performed by comparing cDNA sequences with their corresponding full-length genomic DNA sequences using the online tool Gene Structure Display Server version 2.0 (gsds.cbi.pku.edu.cn/).

### Cis-elements of the PdbNF-Y promoter

The promoter sequences (length, 2 kb) of *PdbNF-Y*s were collected from the *P. davidiana* × *P. bollena* genome database (not made public). The cis-elements were analyzed in the PlantCARE program (https://bioinformatics.psb.ugent.be/webtools/plantcare/html/).

### Protein–protein interaction analyses of PdbNF-Ys

The interaction networks between PdbNF-Ys and other TFs were created by the online software STRING (https://string-db.org/) and visualized by Cytoscape. The intersection database of *P. trichocarpa* was selected as a reference.

### Plant growth and stress treatments

Hybrid poplar seedlings were grown in a greenhouse under 16-h light/8-h dark conditions at 25°C for 15 days. The plant hormone treatments of poplar were carried out as follows: for methyl jasmonate (MeJA) treatment, the seedlings were sprayed with a freshly prepared 0.001% (w/v) MeJA solution; for the salicylic acid (SA) treatment, a new working solution of 5 mM SA was sprayed on the leaves. Leaves from plants were collected at 0, 2, 6, 12, 24, and 48 h after treatment, frozen in liquid nitrogen, and stored at −80°C for RNA extraction. Three independent biological replicates were performed.

The pathogenic fungus *Alternaria alternata* was cultured for 7 days on potato dextrose agar (PDA) medium in the dark and then diluted to 1 × 10^6^ spores/ml with sterile water. Leaves were collected from three-month-old seedlings and inoculated with 10 µl of spore suspension and then placed in a transparent square petri dish with high humidity at 25°C with a 16-h light/8-h dark cycle. Leaves inoculated with sterile water only served as mock controls. The leaves were then sampled at 12, 24, 48, and 72 hpi after inoculation. Three biological replicates, each containing ten parallel leaves from ten different plants, were used for each treatment time point or control. The samples were then rapidly placed in liquid nitrogen and stored at −80°C.

### RNA isolation and quantitative real-time PCR analysis

Total RNA was extracted using the EZNA Plant RNA Kit (R6827, OMEGA, America), and cDNA was synthesized for quantitative real-time PCR (RT‒qPCR) by the TransScript One-Step gDNA Removal and cDNA Synthesis SuperMix Kit (AT311, TransGen Biotech, Beijing, China) according to the manufacturer’s method. RT‒qPCR was carried out using SYBR® Green real-time PCR Master Mix (QPK-201, TOYOBO, OSAKA, Japan) in a CFX96 TouchTM real-time PCR detection system (Bio-Rad, Hercules, CA, USA). The housekeeping genes *PdbEF* and *Pdbactin* were selected as internal controls, and the primers used for RT‒qPCR analysis are listed in Supplementary Table S1. Relative gene expression levels were measured using the 2^−ΔΔCt^ method (Livaka and Schmittgen, 2001). Genes with changes in the expression level greater than 2-fold were considered to be significantly regulated.

### Generation of transgenic plants

The complete ORF of *PdbNF-YA11* was cloned into the *KpnI* and *XbaI* restriction enzyme sites of pROKII under the control of the CaMV 35S promoter and NOS terminator to generate the overexpression construct pROKII-PdbNF-YA11 (*PdbNF-YA11*-OE). A 228-bp truncated inverted repeat CDS of *PdbNF-YA11* was inserted into the RNAi vector pFGC5941 (pFGC::*PdbNF-YA11*) to silence the expression of *PdbNF-YA11* (*PdbNF-YA11*-IE). The primers used are listed in Supplementary Table S1. Then, the construct was introduced into the Agrobacterium strain EHA105, which was then transformed into *P. davidiana* × *P. bollena via* *Agrobacterium*-mediated leaf-disk transformation (Ji et al., 2016). The transgenic plants were selected using kanamycin (50 mg/L) or glyphosate (1 mg/L), and all transgenic plants were verified *via* genomic PCR and RT‒qPCR.

### *Alternaria alternata* resistance assay in transgenic plants

Leaves collected from 1-month-old transgenic plants and wild-type plants were inoculated with 1 × 10^6^/ml *A. alternata* spores and then placed in a transparent square petri dish under the conditions described above. The leaves were observed and sampled 4 d after inoculation, and the leaf lesion areas were measured by using ImageJ software (Geng et al., 2020). The infection ratings were assigned and calculated according to the method of Pandey et al. (2003). Three biological replicates, each containing ten parallel leaves from ten different plants, were used for each treatment time point or control. The sampled leaves were then used to determine the JA contents and the expression of JA-related genes. The JA contents were determined *via* enzyme-linked immunosorbent assay (ELISA) kits (Shanghai, China RJ-21830). The RT‒qPCR followed the method described above, and the primers used for analysis are listed in Supplementary Table S1. For each assay, three biological replicates were analyzed.

### Statistical analyses

Statistical analyses were carried out using SPSS v.20 software. The data were compared using Student’s t test. Differences between the two groups of data were considered statistically significant at *p* < 0.05. * indicates a significant difference.

## Results

### Cloning and sequence analysis of *PdbNF-Y*s in *P. davidiana* × *P. bollena*

A total of 26 *PdbNF-Y* genes (12 *PdbNF-YA*s, six *PdbNF-YB*s, and eight *PdbNF-YC*s) were identified and cloned after the local BLAST and HMMER searches (Table 1, Supplementary Figure S1). The coding sequences (CDSs) of *PdbNF-Y*s ranged in size from 453 (*PdbNF-YB5*) to 1101 (*PdbNF-YA1*) bp with deduced proteins of 217–366 amino acids, showing a wide distribution of *PdbNF-Y* lengths. Among the three subfamilies of PdbNF-Y, the PdbNF-YA lengths (average length 326.25 AA) were the longest, the PdbNF-YC lengths (average length 251.2 AA) were shorter, and the PdbNF-YB lengths (average length 193.5 AA) were the shortest. The isoelectric point (pI) of PdbNF-Ys ranged from 5.04 (PdbNF-YB3) to 9.65 (PdbNF-YC4). All PdbNF-Y proteins received negative GRAVY scores, indicating that they are all hydrophilic proteins. Subcellular localization analysis revealed that most of the NF-Y proteins were predicted to be located in the nucleus, while a few were targeted to the cytoplasm or nucleus.

**TABLE 1:** Characteristics of *PdbNF-Y*s in poplar.

Gene name	CDS (bp)	ORF(aa)	MW(kDa)	Isoelectric point (pI)	GRAVY	Subcellular location
*PdbNF-YA1*	1101	366	39.98	9.46	−0.479	Nucleus
*PdbNF-YA2*	1071	356	39.14	6.47	−1.074	Nucleus
*PdbNF-YA3*	819	272	30.41	9.03	−0.901	Nucleus
*PdbNF-YA4*	861	286	31.81	9.50	−0.843	Nucleus
*PdbNF-YA5*	1098	365	40.27	8.34	−0.627	Nucleus
*PdbNF-YA6*	1026	341	37.26	9.15	−0.681	Nucleus
*PdbNF-YA7*	954	317	34.79	9.10	−0.474	Nucleus
*PdbNF-YA8*	1059	352	38.57	8.34	−0.700	Nucleus
*PdbNF-YA9*	654	217	24.19	9.23	−0.862	Nucleus
*PdbNF-YA10*	1062	353	38.83	8.34	−0.708	Nucleus
*PdbNF-YA11*	1026	341	37.69	9.03	−0.680	Nucleus
*PdbNF-YA12*	1050	349	38.65	8.43	−1.098	Nucleus
*PdbNF-YB1*	594	197	21.28	5.75	−0.806	Nucleus
*PdbNF-YB2*	597	198	20.83	6.24	−0.662	Nucleus
*PdbNF-YB3*	504	167	18.24	5.04	−0.663	Nucleus
*PdbNF-YB4*	675	224	24.89	5.74	−0.709	Nucleus
*PdbNF-YB5*	453	150	16.31	6.60	−0.779	Nucleus
*PdbNF-YB6*	678	225	24.79	6.97	−0.618	Nucleus
*PdbNF-YC1*	753	250	28.08	5.97	−0.559	Cytoplasm. nucleus
*PdbNF-YC2*	741	246	26.81	5.07	−0.407	Cytoplasm. nucleus
*PdbNF-YC3*	777	258	28.83	5.71	−0.498	Nucleus
*PdbNF-YC4*	636	211	23.70	9.65	−0.964	Nucleus
*PdbNF-YC5*	891	296	33.14	5.69	−0.531	Nucleus
*PdbNF-YC6*	843	280	30.83	5.64	−0.365	Nucleus
*PdbNF-YC7*	657	219	24.76	5.87	−0.875	Nucleus
*PdbNF-YC8*	753	250	28.05	5.97	−0.551	Cytoplasm. nucleus

### Multiple alignments of PdbNF-Ys

**FIGURE 1:**
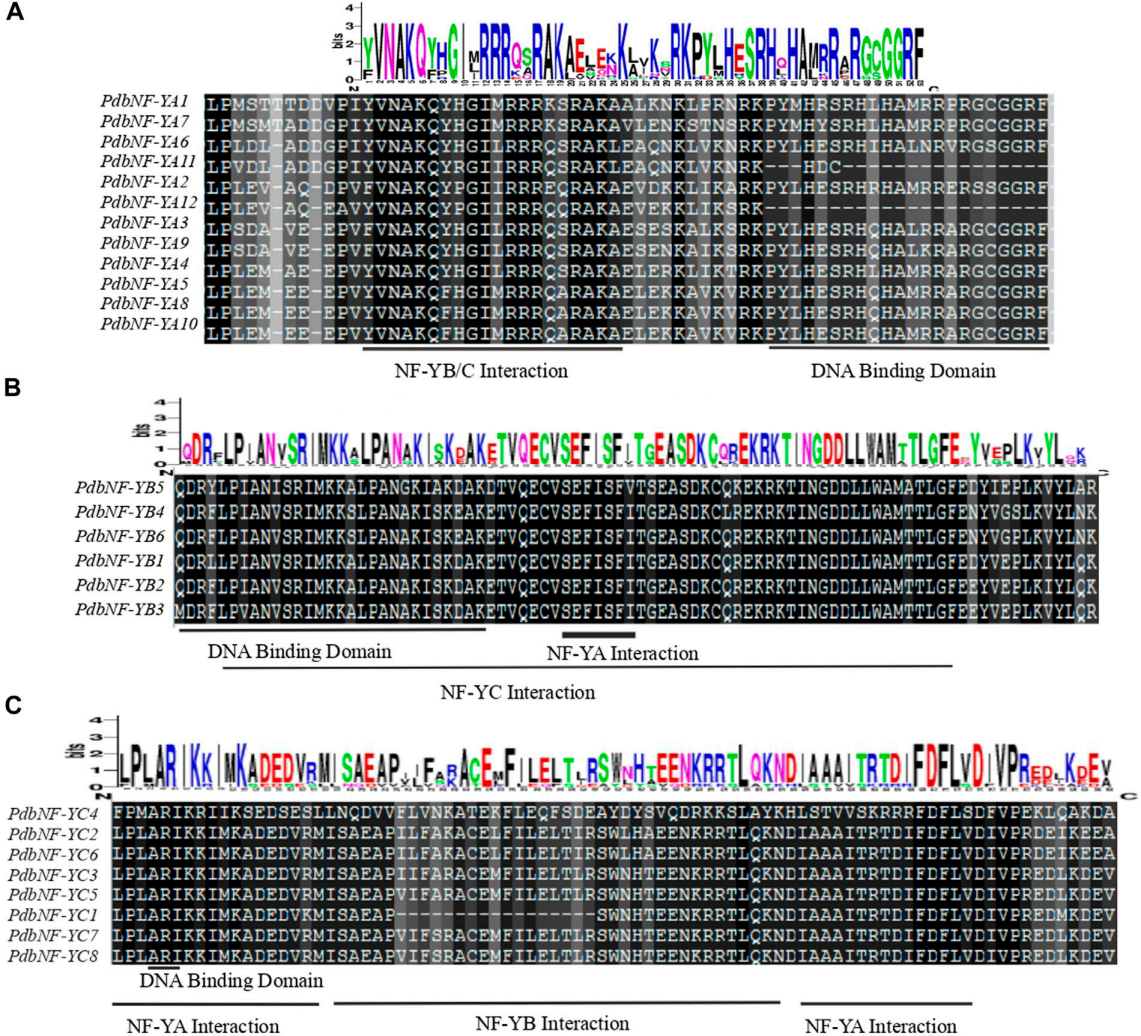
Multiple alignments of the PdbNF-Y family. **(A)** Multiple alignments of the PdbNF-YA conserved domains. **(B)** Multiple alignments of the PdbNF-YB conserved domains. **(C)** Multiple alignments of the PdbNF-YC conserved domains. The logo of the conserved domains was visualized in WebLogo.

Multiple alignment analysis revealed that the amino acid sequences of NF-Ys contain an interaction domain for interacting with other NF-Y subunits and a DNA binding domain for recognizing the *CCAAT* binding sites. The conserved domain of PdbNF-YAs consists of two subdomains involved in the NF-YB/C interaction and DNA binding (Figure 1A). The conserved domain of PdbNF-YBs is composed of a DNA-binding domain and NF-YA/NF-YC interaction domains (Figure 1B). The conserved domain of PdbNF-YCs is characterized by a subdomain (distributed in two separate regions) responsible for NF-YA interaction (Figure 1C) and a DNA-binding domain consisting of only two residues in series “A” and “R.” Furthermore, the logo of PdbNF-Y conserved domain sequences suggested that PdbNF-YBs were more evolutionarily conserved than other types of PdbNF- Y subunits.

### Evolutionary relationships of the NF-Y family in *P. davidiana* × *P. bollena*, *P. trichocarpa*, and *Arabidopsis*

**FIGURE 2:**
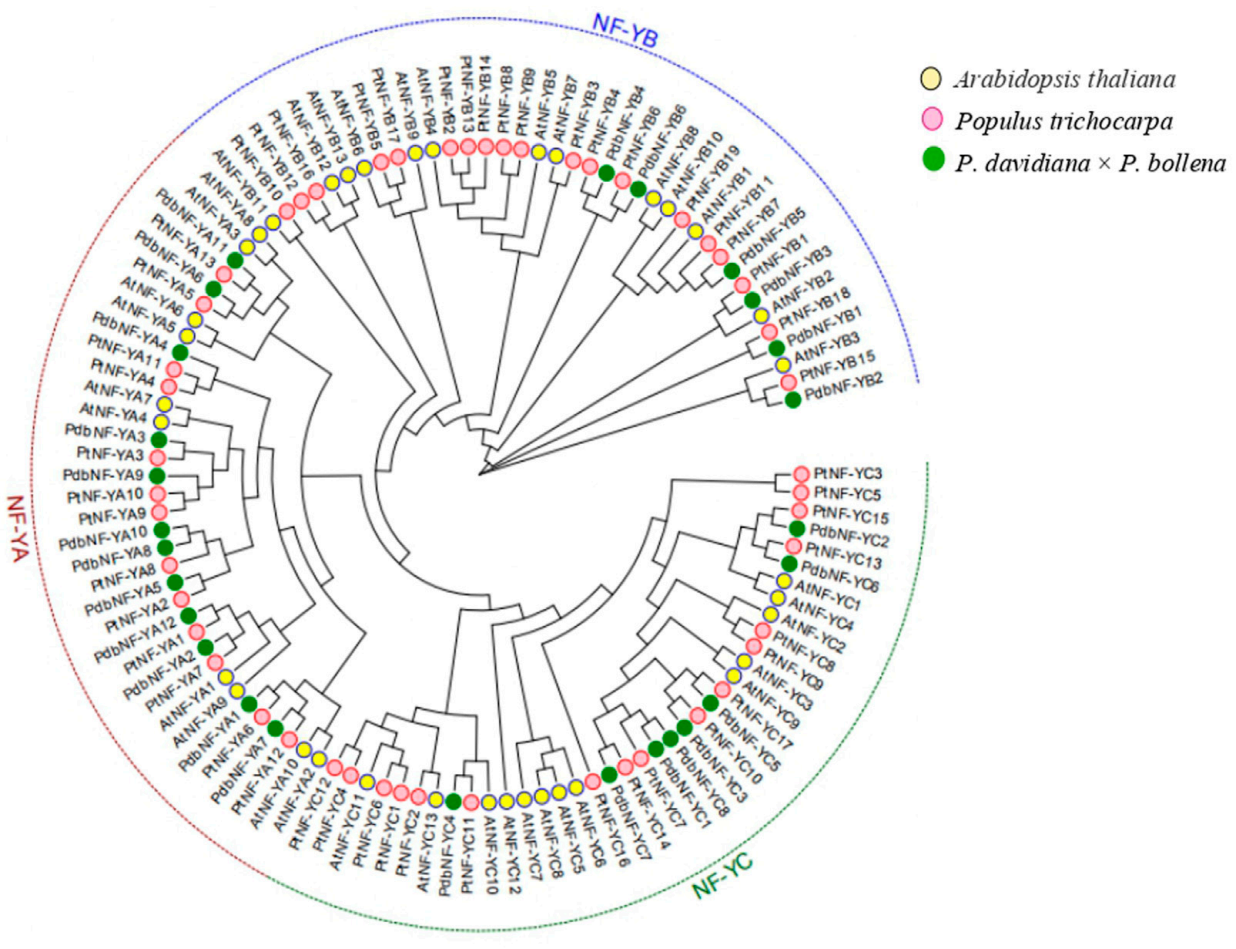
Phylogenetic analysis of the NF-Y TFs in poplar and *Arabidopsis*. All NF-Y TFs were clustered into three clades (NF-YA, NF-YB, and NF-YC).

To further elucidate the evolutionary relationships of the NF-Y family members between poplar and *Arabidopsis*, a phylogenetic tree was constructed with the NF-Y protein sequences of poplar and *Arabidopsis* (Figure 2). The NF-Y proteins were clustered into three clades (NF-YA, NF-YB, and NF-YC). Both the NF-YA and NF-YC groups contained one pair of paralogous NF-Y proteins, while the NF-YB group contained four pairs. The phylogenetic tree showed close relationships between the NF-Y members within each of the three subunits and a close relationship of the NF-Y family members between the two poplars and *Arabidopsis*, which indicated that the NF-Y protein family in poplar may have a structure and function similar to those in *Arabidopsis*.

### Gene structure and conserved motifs of PdbNF-Ys

**FIGURE 3:**
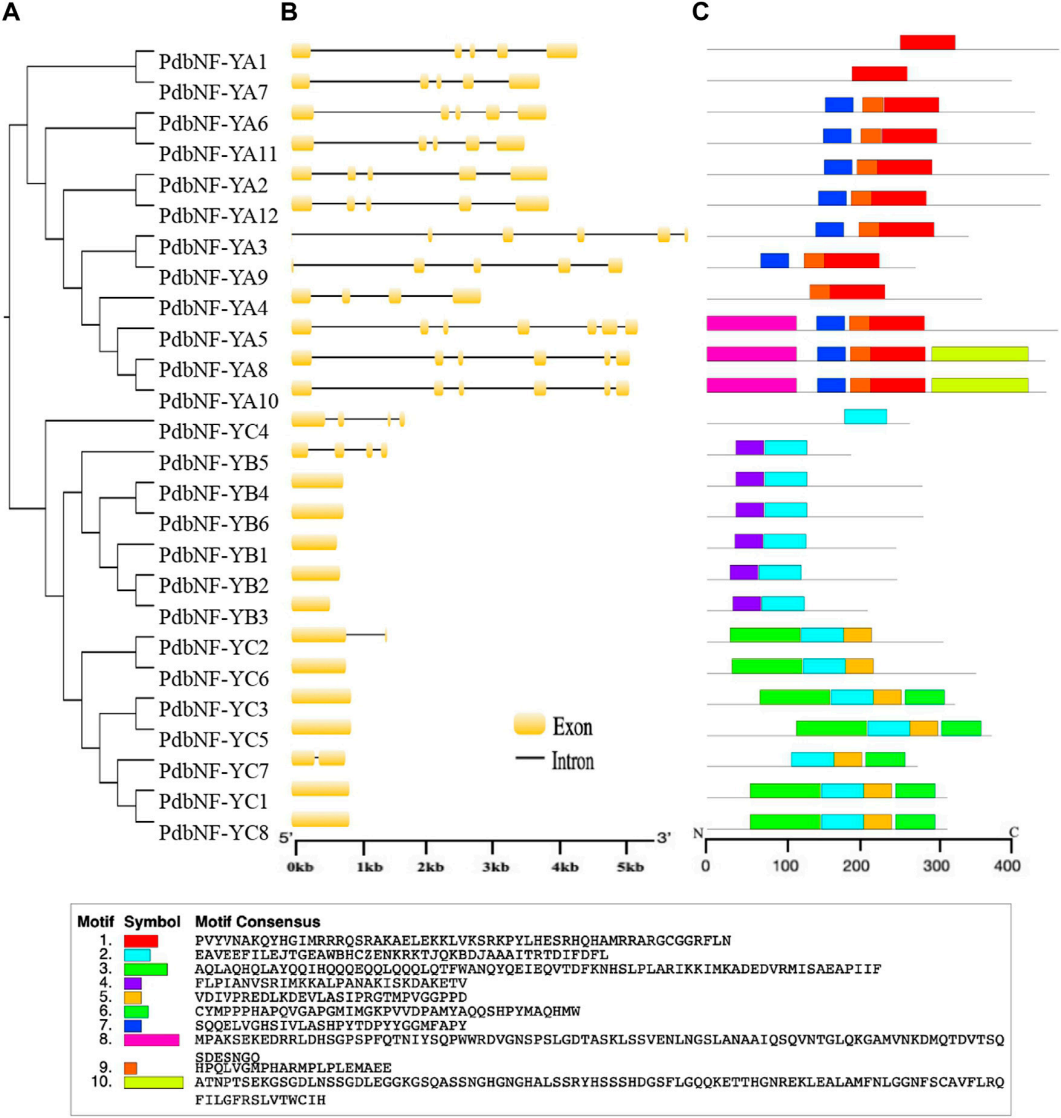
Phylogenetic relationships, gene structures, and motif compositions of the *PdbNF-Y* genes. **(A)** Unrooted NJ tree. **(B)** Exon/intron organization. Exons are shown as yellow boxes, and introns are shown as black lines. **(C)** Schematic representation of conserved MEME motifs of full-length NF-Y proteins. Colored boxes indicate different conserved motifs, while the black lines represent sequences with no MEME motifs detected.

Phylogenetic analysis was performed for PdbNF-Ys, and most of the PdbNF-Ys were clustered in pairs, except PdbNF-YA4, PdbNF-YA5, PdbNF-YB1, PdbNF-YB5, PdbNF-YC4, and PdbNF-YC7 (Figure 3A). These paired genes also shared similar gene structures, including the numbers and positions of both introns and exons (Figure 3B). Among these, the *PdbNF-YA* subfamily contained a larger number of introns (from four to six); one or three introns were found in the *PdbNF-YC*, while most of the *PdbNF-YB*s contained no introns, except *PdbNF-YB5* (Figure 3B). The conserved motifs in PdbNF-Y proteins were analyzed by MEME, and a total of 10 motifs were identified. The results showed that all the PdbNF-Ys contained motifs involved in interacting with the other two NF-Y subfamilies and DNA binding. In addition, motif 9 was identified in most of the PdbNF-YAs, except PdbNF-YA1 and PdbNF-YA7, and motifs 8 and 10 were observed only in PdbNF-YA5/8/10 and PdbNF-YA8/10, respectively (Figure 3C).

### Analysis of cis-elements in *PdbNF-Y* promoters

**FIGURE 4:**
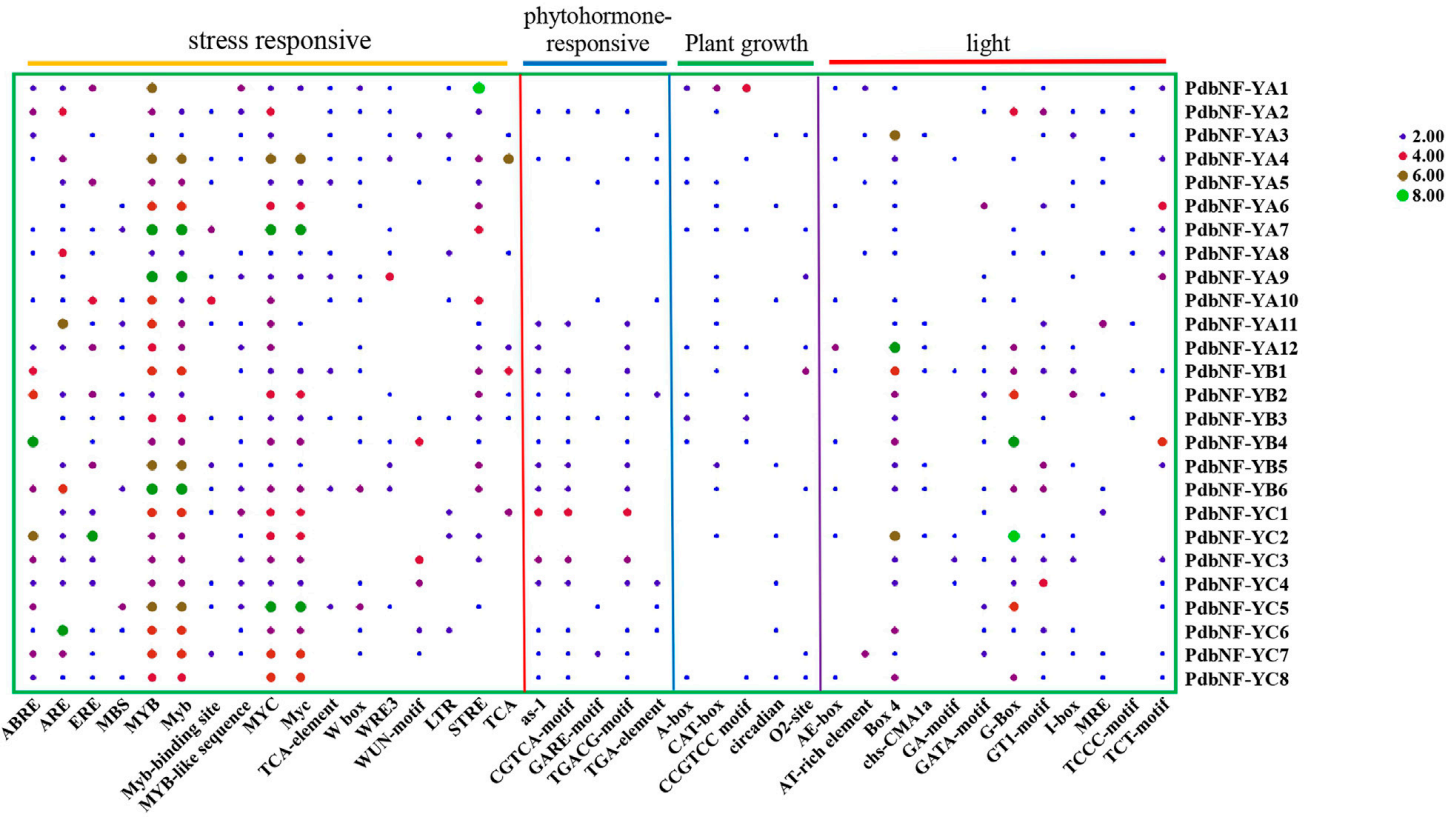
Heatmap of cis-elements in the promoter regions of the *PdbNF-Y*s. Colored bars indicate the numbers of cis-elements.

To explore the potential function and regulation of *PdbNF-Y* genes, the 2000 bp upstream sequences of their promoters were analyzed. A total of 39 cis-elements were found, including 12 light responsive, five phytohormone-responsive, five plant growth-responsive, and 17 stress-responsive cis-elements (Figure 4). Among the cis-elements, stress-responsive elements accounted for the largest proportion. Several elements related to disease resistance were found among the stress-responsive elements. For example, MYC is related to JA signaling; W-box can bind to WRKY, which plays roles in plant disease resistance; and MYB is also related to disease response. Analysis of the phytohormone-responsive elements indicated that the TGA element is involved in auxin responsiveness, GARE motif is involved in gibberellin responsiveness, and TGACG-motif and CGTCA-motif are involved in MeJA responsiveness. In summary, the aforementioned results indicate the potential role of *PdbNF-Y* genes in response to the regulation of a variety of hormones and disease resistance.

### Protein–protein interaction analyses of PdbNF-Ys

**FIGURE 5:**
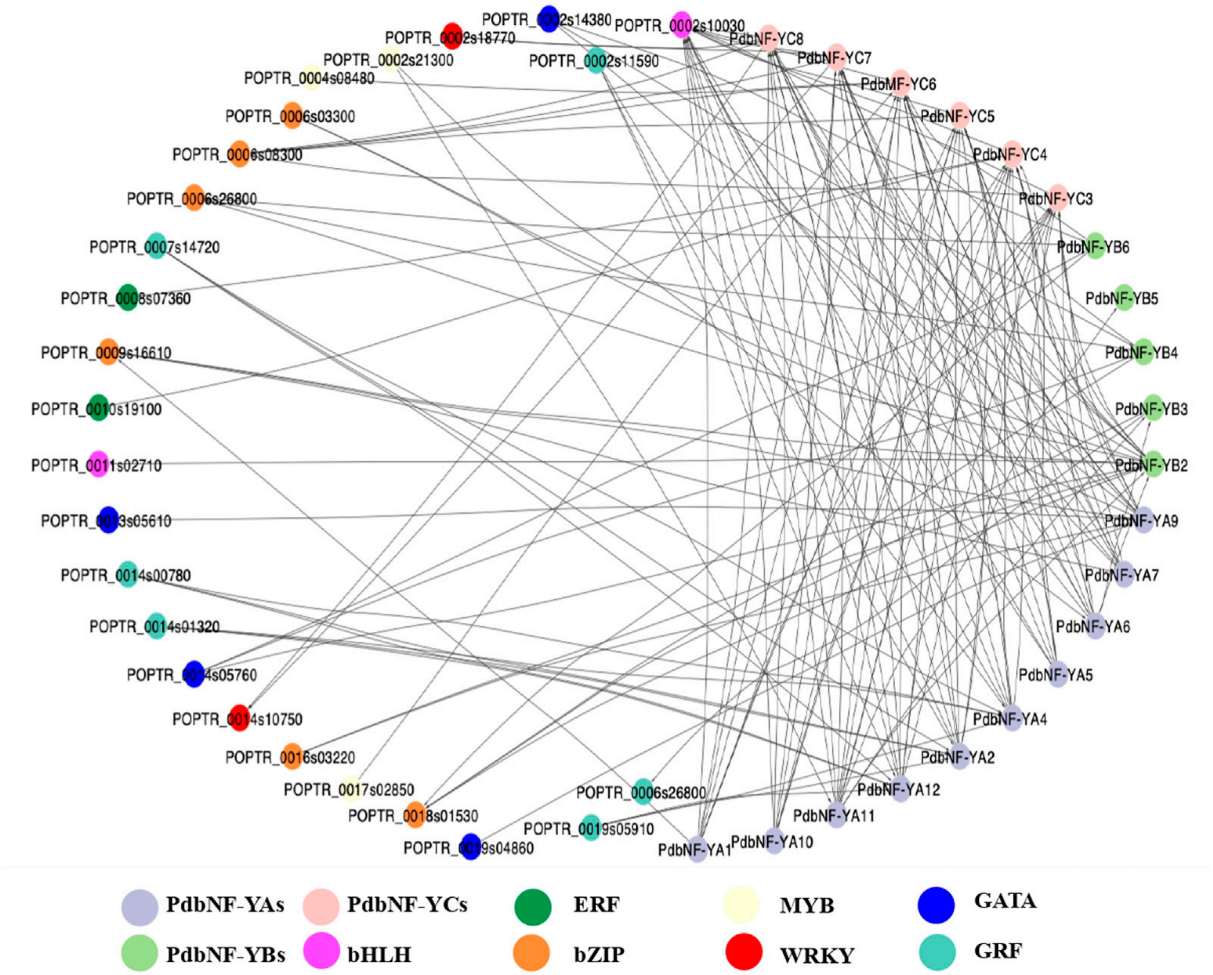
Protein–protein interaction analysis of PdbNF-Ys and other transcription factors. Protein–protein interaction network of PdbNF-Ys and other transcription factors. Colored circles indicate different transcription factors (TFs).

To obtain a more complete overview of the transcriptional regulation function of PdbNF-Ys, a protein–protein interaction (PPI) network was constructed. The results showed that the three PdbNF-Y subunits may interact with the NF-Y proteins belonging to the other two subfamilies, among which the interactions in PdbNF-YB2/PdbNF-YCs (PdbNF-YC3–5, PdbNF-YC7, and PdbNF-YC8), PdbNF-YB2/PdbNF-YAs (PdbNF-YA6 and PdbNF-YA11), and PdbNF-YC4/PdbNF-YB3 had combined scores higher than 0.95 (Figure 5, Supplementary Table S2). Moreover, these PdbNF-Ys can also interact with other TFs. For example, PdbNF-YAs may interact with TFs, such as GATA, GRF, MYB, bHLH, and bZIP; PdbNF-YBs may interact with bHLH and bZIP; and PdbNF-YCs may interact with TFs, including ERF, MYB, WRKY, bHLH, and bZIP (Figure 5). In addition, GO enrichment showed that the aforementioned TFs were primarily involved in “regulation of transcription,” “regulation of molecular function,” “DNA binding,” “CCAAT-binding factor complex,” and so on (Supplementary Table S3).

### Expression patterns of *PdbNF-Y*s in different tissues

**FIGURE 6:**
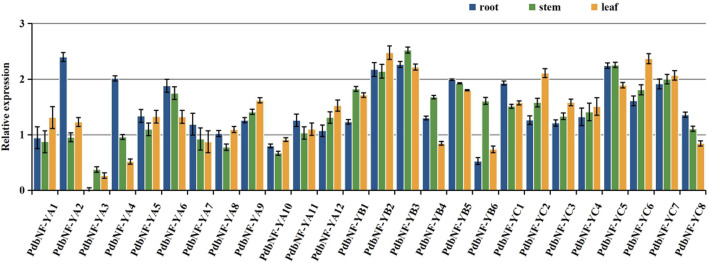
Expression profiles of *PdbNF-Y*s in three poplar tissues. *X*-axis: different PdbNF-Ys and *Y*-axis: expression level = log_2_ (fold change in expression).

Since studies have already shown that *NF-Y*s play roles in regulating plant growth, the expression patterns of *PdbNF-Y*s in vegetative (root, stem, and leaf) tissues were analyzed by RT‒qPCR (Figure 6). As the expression of *PdbNF-YA3* in roots was lower than that of the other genes in all three tissues, we chose its expression as a control for normalization of gene expression in these tissues. For the *PdbNF-YA*s, six of them (*PdbNF-YA2*, *PdbNF-YA4–7*, and *PdbNF-YA11*) had higher transcript levels in roots than in both stems and leaves, and five of them (*PdbNF-YA1*, *PdbNF-YA8–10*, and *PdbNF-YA12*) had higher transcript levels in leaves than in stems and roots, while the expression of *PdbNF-YA3* was higher in stem than in roots and leaves. Among them, *PdbNF-YA2* had the highest transcript level in roots, *PdbNF-YA9* had the highest transcript level in leaves, and *PdbNF-YA6* had the highest transcript level in stems. For the *PdbNF-YB*s, four of them (*PdbNF-YB1*, *PdbNF-YB3*, *PdbNF-YB4*, and *PdbNF-YB6*) had higher transcript levels in stems than in roots and leaves, especially *PdbNF-YB3*, which also had high expression in roots and leaves. However, the expression of *PdbNF-YB2* in leaves was higher than that in roots and leaves, and *PdbNF-YB5* had high expression in roots. For the *PdbNF-YC*s, three of them (*PdbNF-YC1*, *PdbNF-YC5*, and *PdbNF-YC8*) had higher transcript levels in roots than in stems and leaves, while the expression levels of the other *PdbNF-YC*s were higher in leaves than in stems and roots. These results showed tissue-specific *PdbNF-Y*s expression.

### Expression patterns of *PdbNF-Y*s in response to *Alternaria alternata* and hormone treatments

**FIGURE 7:**
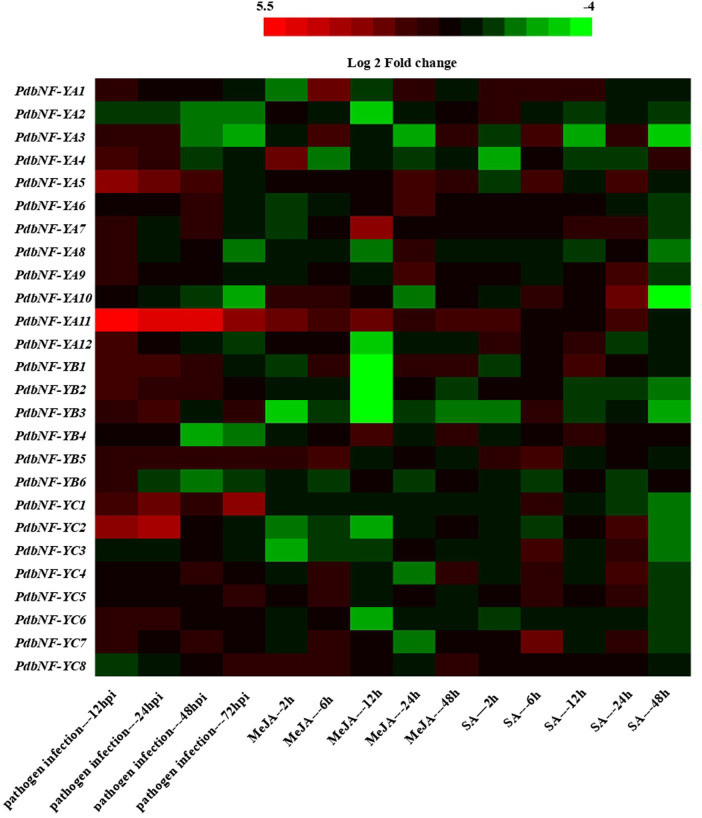
Heatmap of *PdbNF-Y*s expression under biotic stresses. The log_2_ fold change was colored using Cluster 3.0 (red for upregulated and green for downregulated). Each horizontal row represents a gene, and the vertical columns represent time points.

To investigate the transcription of *PdbNF-Y*s in response to plant hormones, RT‒qPCR was performed (Figure 7). Genes with changes in the expression level greater than 2-fold were considered to be significantly regulated. The transcription of *PdbNF-Y*s was induced by exogenous MeJA at different time points in leaves. For the *PdbNF-YA*s, three (*PdbNFYA2–4*) were induced soon after the treatment, but their expressions were subsequently downregulated, while four (*PdbNFYA6–9*) were inhibited after treatment, but their expressions were subsequently upregulated. *PdbNF-YA5* and *PdbNF-YA11* were continuously upregulated, with peak transcription levels of 3.46 fold at 24 h and 4.44 fold at 12 h, respectively. For the *PdbNF-YB*s, two (*PdbNF-YB2* and *PdbNF-YB3*) were continuously downregulated after treatment, while *PdbNF-YB5* was continuously upregulated, with peak transcription levels of 3.56 fold at 6 h. For the *PdbNF-YC*s, three (*PdbNF-YC1–3*) were inhibited after treatment, while three (*PdbNF-YC5-7*) were induced but then downregulated. *PdbNF-YC8* was upregulated and peaked at 6 h.

The transcription levels of the *PdbNF-Y*s were also induced by exogenous SA at different time points in leaves, and most of them were downregulated at 48 h, except *PdbNF-YA4* and *PdbNF-YB6*. For the *PdbNF-YA*s, seven (*PdbNF-YA1*, *PdbNF-YA2*, *PdbNF-YA6*, *PdbNF-YA7*, *PdbNF-YA9*, *PdbNF-YA11*, and *PdbNF-YA12*) were induced soon after the treatment, but their expressions were subsequently downregulated, while the others were upregulated at 24 h after treatment. For the *PdbNF-YB*s, *PdbNF-YB5* was continuously upregulated after treatment, with peak transcription levels of 5.03 fold at 6 h, while *PdbNF-YB2* was inhibited under SA treatment. For the other *PdbNF-YB*s, *PdbNF-YB1*, *PdbNF-YB3*, and *PdbNF-YB4* had the same expression patterns. Among the *PdbNF-YC*s, *PdbNF-YC6* was inhibited after treatment, while four (*PdbNF-YC1*, *PdbNF-YC4*, *PdbNF-YC5*, and *PdbNF-YC7*) were upregulated after 6 h (Figure 7).

The expression of *PdbNF-Y*s after inoculation with the pathogen *A. alternata* was also determined (Figure 7). The RT‒qPCR showed that most of the *PdbNF-YA*s were induced after treatment, except *PdbNF-YA2*. Among these *PdbNF-YA*s, *PdbNF-YA11* was significantly expressed during the treatment, while *PdbNF-YA1*,3–5 and *PdbNF-YA9*,12 had high transcript levels at 12 hpi, but their expressions were subsequently downregulated. For the other *PdbNF-YA*s, their expressions were repressed or not obviously induced at different time points. The *PdbNF-YB*s, including *PdbNF-YB1-2* and *PdbNF-YB6*, were induced at 12 hpi, and their expression levels were downregulated later, while the expression of *PdbNF-YB5* was upregulated during the treatments. For the *PdbNF-YC*s, *PdbNF-YC1* was induced after inoculation, and the expression levels of *PdbNF-YC2* and *PdbNF-YC6* were upregulated at early stages of treatment (12 and 24 hpi), while two *PdbNF-YC*s (*PdbNF-YC3* and *PdbNF-YC8*) were inhibited at 12 and 24 hpi. These results indicate that *PdbNF-Y*s may be involved in different stages of progression during poplar interaction with *A. alternata*. Moreover, JA and SA, the main hormones related to plant disease resistance, are considered the most important signaling molecules in plant defense responses. The upregulation of marker genes involved in the two pathways indicates the activation of these pathways. The expression profiles of marker genes belonging to the JA and SA pathway were also determined by RT‒qPCR, and gene cluster analysis was performed to illustrate the putative molecular mechanism by which the *PdbNF-Y*s respond to pathogen infection (Supplementary Figure S2). The result showed that 15 *PdbNF-Y*s, including nine *PdbNF-YA*s, four *PdbNF-YB*s, and two *PdbNF-YC*s, had the same expression profiles as the genes involved in the JA signaling, while the others had the same expression profiles as the genes involved in the SA signaling.

In brief, the transcription of the *PdbNF-Y*s was affected by all the treatments. Both plant hormone and plant pathogenic fungus treatments could trigger *PdbNF-Y*s transcription.

### *PdbNF-YA11* enhances the resistance of *P. davidiana* × *P. bollena* to *A. alternata* by modulating the plant hormone signal pathway

**FIGURE 8:**
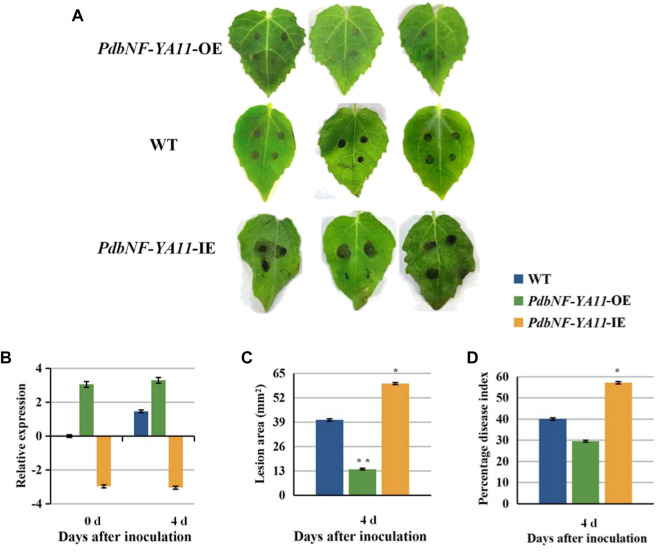
*PdbNF-YA11* enhances resistance to *A. alternata* in poplar. **(A)** Leaves of WT and transgenic poplar inoculated with *A. alternata* plugs for 4 dpi. **(B)** Relative expression levels of *PdbNF-YA11* during infection in WT and transgenic plants. **(C)** Lesion areas of leaves. **(D)** Percentage disease index at 4 dpi. The error bars represent the standard deviation of triplicate assays. Asterisks show significant differences between inoculated and noninoculated leaves (**p < 0.01 and *p < 0.05).

**FIGURE 9:**
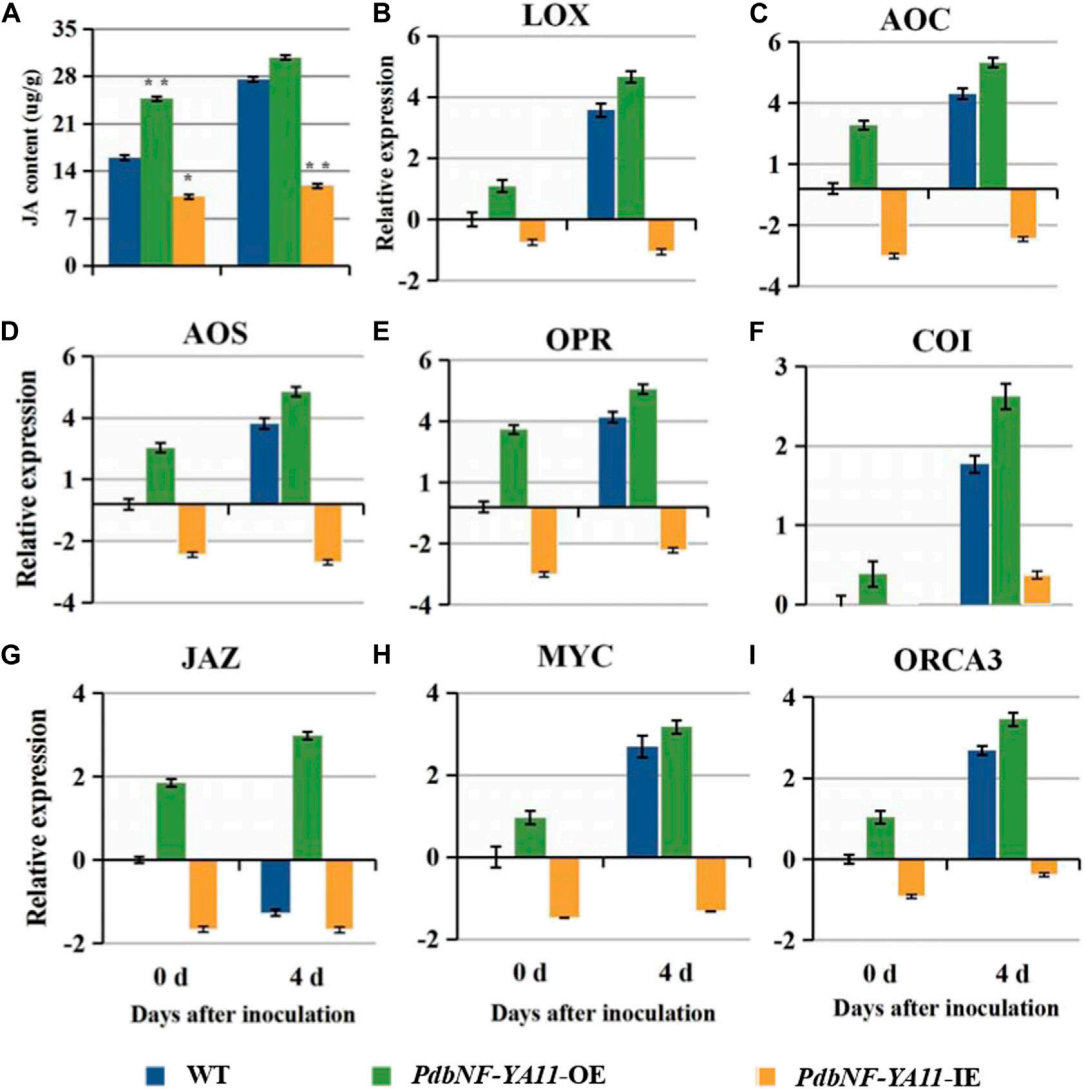
*PdbNF-YA11* regulates JA signaling. **(A)** JA contents in WT and transformed plants. **(B)**–**(I)** Relative expression levels of the JA biosynthetic and JA signaling pathway genes. The error bars represent the standard deviation of triplicate assays. Asterisks show significant differences between inoculated and noninoculated leaves (**p < 0.01 and *p < 0.05).

Based on the expression analysis aforementioned, *PdbNF-YA11* was chosen for further study of its role in poplar disease resistance. The *PdbNF-YA11*-overexpressing (*PdbNF-YA11*-OE) and RNAi-silenced *PdbNF-YA11* (*PdbNF-YA11*-IE) plants were obtained *via* *Agrobacterium* infection (Figure 8B). To examine whether *PdbNF-YA11* affects leaf rot during *A. alternata* infection, intact leaves obtained from the transgenic lines and the corresponding wild-type (WT) parental lines were spot-inoculated with conidia. The results showed that the severity of tissue damage upon *A. alternata* infection was significantly lower in the leaves of overexpression poplars than in their WT siblings at 4 d post inoculation (dpi), while the damage severity was higher in RNAi poplars, further confirming the percentage disease index results (Figures 8A–D). In addition, as the expression of *PdbNF-YA11* was induced under MeJA treatment, the expression profiles of genes related to JA synthesis and signaling were analyzed in transgenic plants by RT‒qPCR, and the JA contents were also analyzed during infection. These genes showed significantly higher relative expression levels than those in the WT during infection in *PdbNF-YA11*-OE plants, while the expression levels of these genes were downregulated in *PdbNF-YA11*-IE plants (Figures 9B–I). Meanwhile, the JA contents in the overexpression lines were higher than those in the WT during infection but were lower in the *PdbNF-YA11*-IE lines than the WT (Figure 9A). These results implied that *PdbNF-YA11* is involved in the resistance of poplar to *A. alternata* infection by regulating the JA synthesis and signaling pathways.

## Discussion

As a transcription factor (TF) that regulates plant growth and stress resistance, the NF-Y has been identified and studied in multiple plants. There were 36 NF-Ys in *A. thaliana* (Siefers et al., 2009), 25 NF-Ys in *Ricinus communi* (Wang et al., 2018), 59 NF-Ys in *Solanum lycopersicum* (Li et al., 2016), 32 NF-Ys in *Vitis vinifera* (Ren et al., 2016), 33 NF-Ys in *Juglans regia* (Quan et al., 2018), 24 NF-Ys in peach (Li et al., 2019), 28 NF-Ys in *Pinus tabuliformis* (Guo et al., 2020), 52 NF-Ys in *P. trichocarpa* (Liu et al., 2021), and 46 NF-Ys in *P. tomentosa* (Li et al., 2022). In this study, a total of 26 NF-Y members were cloned in *P. davidiana* × *P. bollena*, including 12 *PdbNF-YA*s, six *PdbNF-YB*s, and eight *PdbNF-YC*s (Table 1, Supplementary Figure S1). The reason for the low numbers of *NF-Y* genes identified in *P. davidiana* × *P. bollena* may be associated with the lack of a high-quality genome assembly, which may have resulted in some *NF-Y* family genes not being successfully cloned. The *NF-Y*s are well known to be involved in plant growth and physiology. However, few studies of the *NF-Y* family concerning biotic stress resistance have been performed in poplar. As such, we further analyzed the function of *PdbNF-Y*s involved in biotic stress response, providing a valuable resource for further functional research on *NF-Y* genes in *Populus*.

In *Arabidopsis* and *P. trichocarpa*, the biological functions of some *NF-Y*s have been identified. For example, *PtNF-YA9* was shown to be involved in abiotic stress and to alter leaf size by mediating the expression of auxin-related genes (Lian et al., 2018). Both *AtNF-YB2* and *AtNF-YB3* were shown to be involved in root development and flowering time regulation (Kumimoto et al., 2008). *AtNF-YC3*, *-4*, and *-9* have been proven to control flowering time (Kumimoto et al., 2010), and *NF-YB9* has been shown to be a pivotal regulator in embryogenesis (Siefers et al., 2009). In this study, phylogenetic relationships between *Arabidopsis* and poplar were examined, and the high sequence identity between them can be used to infer similar functions. The results showed that *PdbNF-YA9* is homologous to *PtNF-YA9* (Figure 2) and had a high transcript level in leaves (Figure 6), suggesting that it may be involved in regulation of leaf development in *P. davidiana* × *P. bollena*. Moreover, *PdbNF-YB3* and *PdbNF-YB2* showed homology to *AtNF-YB2* and *AtNF-YB3*, respectively (Figure 2), which both have high transcript levels in three tissues (Figure 6) and may have similar roles in poplar.

Recently, *NF-Y*s have been shown to play key roles in plants in response to stress resistance. For example, *NF-YA*s from *Arabidopsis*, *StNF-YC9* from potato, and *OsNF-YA7* from rice were proven to participate in drought resistance (Leyva-González et al., 2012; Dong-Keun et al., 2015; Li et al., 2021). Both *CdtNF-YC1* from *B. grass* and *TaNF-YA10–1* from wheat play roles in drought and salinity tolerance (Chen et al., 2015; Ma et al., 2015). *GmNFYA* may regulate salt tolerance in soybean (Lu et al., 2021). *ZmNF-YA3* and *ZmNF-YA8* in maize were upregulated after infection by the leaf pathogens, *S. reiliana* and *C. graminicola* (Zhang et al., 2016). *OsHAP2E* conferred resistance to *M. oryzae* and *X. oryzae* pv. *oryzae *in rice by regulating defense- and signaling-related genes (Alam et al., 2015). The NF-Y in cassava may play roles in the plant defense response by regulating *MePR* (He et al., 2020). In poplar, *PtNF-YA9* and *PtNF-YB7* were proven to regulate resistance to abiotic stress in transgenic *Arabidopsis* (Han et al., 2013; Lian et al., 2018), but little is known about the roles of *NF-Y*s during disease resistance. In this study, cis-element analysis showed that the *PdbNF-Y* promoters contain elements related to disease resistance, such as MYC, W-box, and MYB (Figure 4), and RT-qPCR showed that most of the *PdbNF-Y*s were significantly changed during the *A. alternata* infection (Figure 7). Further analysis also showed that these PdbNF-Ys have the same expression profiles as the genes involved in SA/JA signaling, which are known to be involved in disease resistance (Supplementary Figure S2). These results proved that *PdbNF-Y*s may be involved in poplar disease resistance.

Phytohormones, especially jasmonates (JAs) act as signals to trigger and mediate a diverse array of defense responses. Transcription factors (TFs) such as MYC2, ERF, and WRKY have been shown to be involved in plant disease resistance by regulating the hormone signaling pathway. For example, MYC2 and its downstream MYC2-targeted TFs (MTFs) form a transcription module that may directly regulate the JA-induced transcription of late defense genes during tomato resistance to *B. cinerea* (Du et al., 2017). *WRKY75* could also positively regulate the JA-mediated plant defense against *B. cinerea* and *A. brassicicola* by regulating genes such as *ORA59* and *PDF1.2* in *Arabidopsis* (Chen et al., 2020). In *Pinus tabuliformis*, the *NF-Y*s were shown to respond to SA and JA treatments, which suggests their roles in affecting disease resistance by dynamically regulating SA and JA signaling (Guo et al., 2020). In this study, the *PdbNF-Y* promoters contain elements related to hormone response (Figure 4), and the protein–protein interaction (PPI) network showed that the PdbNF-Ys may interact with TFs involved in plant hormone signal transduction, such as ERF, MYB, WRKY, and bHLH (Figure 5, Supplementary Table S3). Importantly, the expression levels of *PdbNF-Y*s were significantly changed under phytohormone treatments (Figure 7), which indicates that these *PdbNF-Y*s may be involved in hormone-mediated regulation of defense responses.

Based on the results aforementioned, *PdbNF-YA11* contains multiple types of disease-responsive cis-elements in its promoter, and its expression under pathogen infection and hormone treatments was significantly induced. As such, *PdbNF-YA11* was chosen for further study of its role during plant‒pathogen interactions in leaves. Both the *PdbNF-YA11*-overexpressing (OE) and *PdbNF-YA11* RNAi-silenced (IE) poplars were generated by *Agrobacterium*-mediated leaf-disk transformation, and the leaves were observed after inoculation with *A. alternata*. The *PdbNF-YA11*-OE plants exhibited significantly higher resistance to *A. alternata* infection than WT plants, while the *PdbNFYA11*-IE lines were more susceptible to *A. alternata* than WT plants (Figure 8). Moreover, JA is commonly known to be involved in plant defense mechanisms against necrotrophic pathogens, such as *Alternaria*, *Botrytis cinerea*, *Sclerotinia sclerotiorum*, *Plectosphaerella cucumerina*, *Fusarium oxysporum*, and *Pythium* (Glazebrook et al., 2005). In this study, the expression levels of JA-related gene and JA accumulation were increased in the *PdbNF-YA11*-OE plants, but the opposite effects were observed in the *PdbNF-YA11*-IE plants during infection (Figure 9). Overall, these results proved that *PdbNFYA11* may be involved in poplar resistance to pathogen infection by positively regulating the JA-related pathway.

In this study, 26 *PdbNF-Y*s were cloned from the hybrid poplar *P. davidiana* × *P. bollena*. Specific information on the protein architecture was determined, and phylogenetic analysis was performed. Then, the expression profiles of the genes were detected in diverse poplar tissues and seedlings under different treatments. Additionally, *PdbNF-YA11* was shown to be involved in the resistance of poplar to *A. alternata* infection by regulating JA synthesis and signaling pathways by genetic engineering. These results suggested that *PdbNF-Y*s may be essential not only for plant development but also for the response to biotic stress and provide a basis for further studies on the functions of the *PdbNF-Y* family.

## Data Availability

The original contributions presented in the study are included in the article/Supplementary Material; further inquiries can be directed to the corresponding author.
