# Supplementary figures and images for: Expression patterns of the poplar NF-Y gene family in response to Alternaria alternata and hormone treatment and the role of PdbNF-YA11 in disease resistance
Source: Front Bioeng Biotechnol. 2022 Sep 16;10:956271. doi: 10.3389/fbioe.2022.956271 (PMC9523018; doi:10.3389/fbioe.2022.956271)

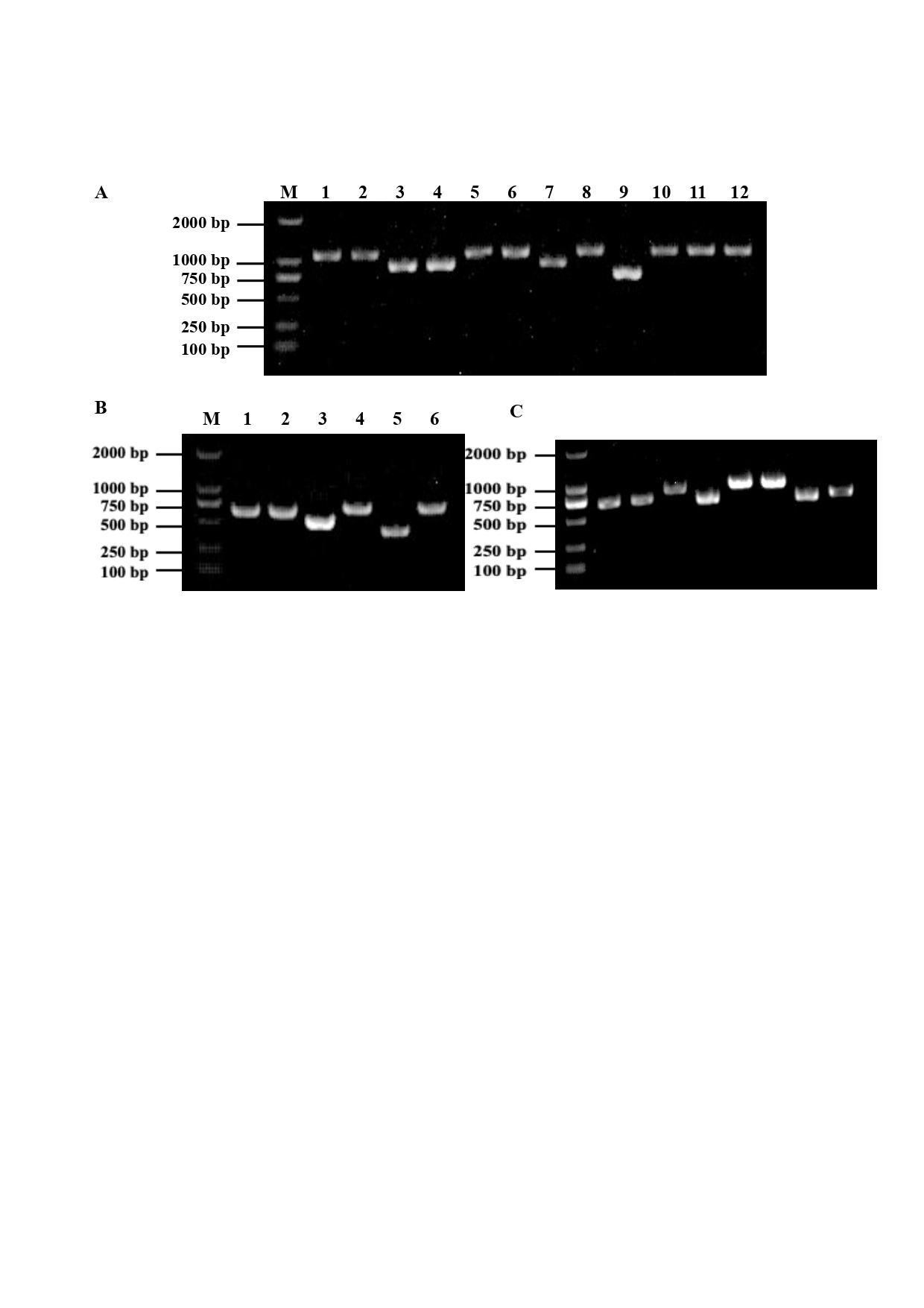

Supplement: Supplementary file 1 [file Image1.JPEG]

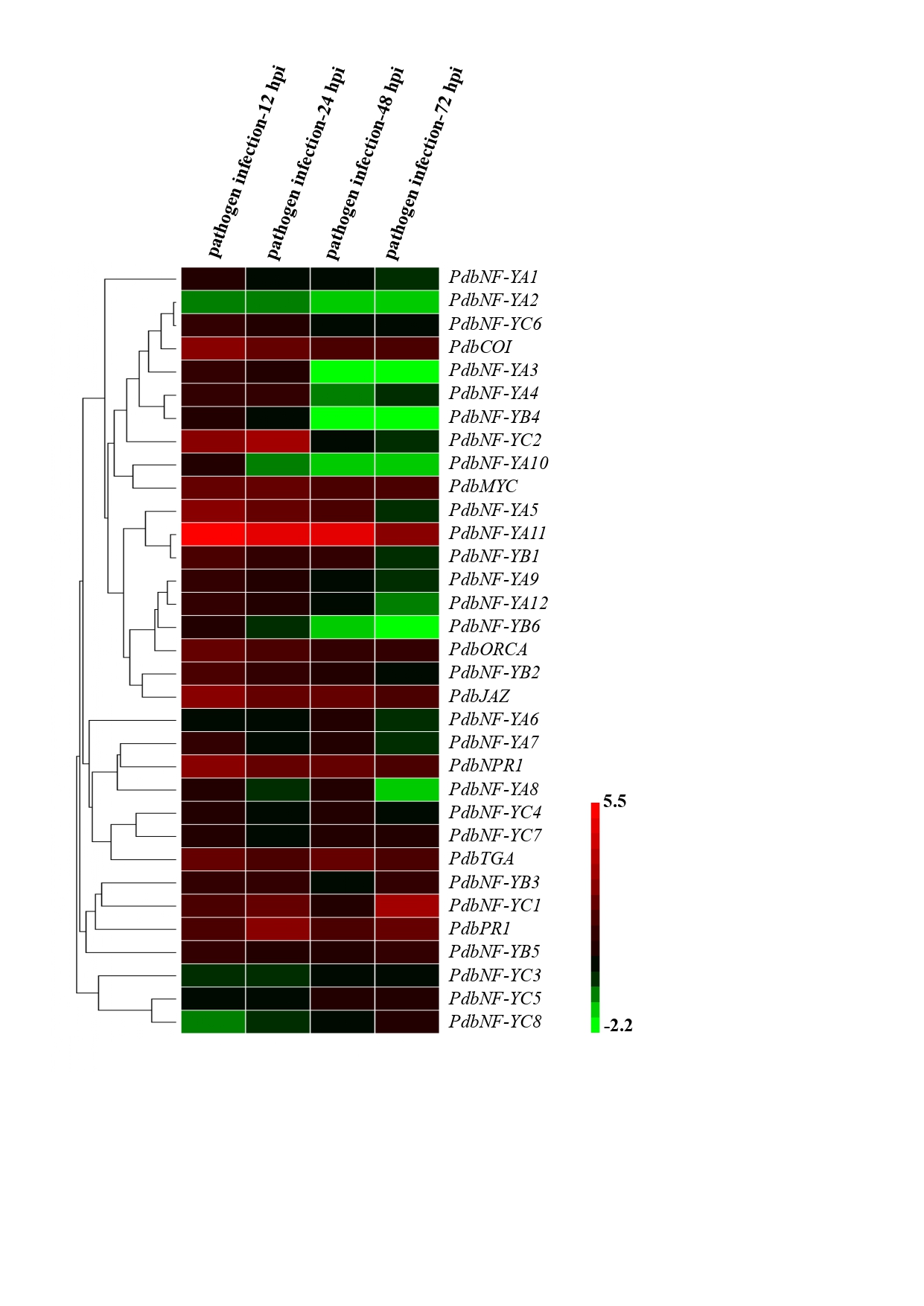

Supplement: Supplementary file 2 [file Image2.JPEG]
