# Supplementary material for: Expression patterns of the poplar NF-Y gene family in response to Alternaria alternata and hormone treatment and the role of PdbNF-YA11 in disease resistance
Source: Front Bioeng Biotechnol. 2022 Sep 16;10:956271. doi: 10.3389/fbioe.2022.956271 (PMC9523018; doi:10.3389/fbioe.2022.956271)
Supplement: Supplementary file 4 [file DataSheet1.docx]

Table S1 Primers used for RT-qPCR

| Genes | Forward and reverse primers(5’-3’) | |
| --- | --- | --- |
| *PdbNF-YA1* | TGCTTCACCCGTAGAACAGC | ACCACCTGAAGGTTTGCAGT |
| *PdbNF-YA2* | TTCACTCGGAACCTTGGTGG | TGTGAGAGGCCATTGTGCAT |
| *PdbNF-YA3* | GCCCTATGGTGCACAACCGAT | CAGTGCATGCTGATGTCGAG |
| *PdbNF-YA4* | AGGGACCAAGGCCATCAAAC | GTCTTCGCCTCAGAATGCCA |
| *PdbNF-YA5* | GCGTGAAGTGGGGAACAATC | GGTTCAAGGTGTCCGTCCAT |
| *PdbNF-YA6* | TGGACGCTTTCTCAGCACAA | TGATTTGCACACCTCCACCA |
| *PdbNF-YA7* | TGAAGCTGCGACTAAGCAGG | TCCATCATCTGCGGTCATGC |
| *PdbNF-YA8* | GGTCACAGGCTTCCTCCAAT | CTCACCACCATGAATGCACC |
| *PdbNF-YA9* | CCAGGCATCTCACCTCCAAAT | CGAGATTGCCGTCGTCTCAA |
| *PdbNF-YA10* | TTACTCCCAACCTTGGTGGC | CCGCCCATGGTAACAGTTGT |
| *PdbNF-YA11* | TGGTCCACTTCGTGCATCTC | GCAGAAGACGTTTTGGCCTG |
| *PdbNF-YA12* | GGTGGAATTCAAAGGAACAGCA | TCATGCCCTTCCCACAACTT |
| *PdbNF-YB1* | GTTAGGCAAGGCGATCAAAG | AGCTCCACTACCAGGACCAT |
| *PdbNF-YB2* | TACGTGGAGCCCCTGAAGAT | ACCGTACACGTGTCCTTGAT |
| *PdbNF-YB3* | CGGTGATGATCTCTTGTGGG | AAAACCCAGCACCAGAGCCG |
| *PdbNF-YB4* | CCATGGCACTGTCGGTACTC | ATCGCAATTCCTGTCTCCGT |
| *PdbNF-YB5* | TTACCAGCGAGGCGAGTGAT | TCCAACTGCATCCCGTTTAG |
| *PdbNF-YB6* | TGGCTAGACAAGAGGACCAGT | GCCATTCCGGTCTCCATGAT |
| *PdbNF-YC1* | GGAACAAGTCCGTATCAGCC | CCTAGCCAAGGGAAGGCTATG |
| *PdbNF-YC2* | CTTGGTGGAATTGTCGGTGC | TCCCTTGTCCACCACTAGCA |
| *PdbNF-YC3* | CAGCAGTAGGCATGGTTGGA | CTAGCCAAGGGCAGGCTATG |
| *PdbNF-YC4* | GAGAAGAAGATGGGACGGCG | CCAACGCATCCTTCGCTTG |
| *PdbNF-YC5* | TGAGGTGTTAGCATCAGTCCC | TTTGCTGTGGCCAAATCTGC |
| *PdbNF-YC6* | GGTGGAATTGTCGGCGCTA | ACCCTTGTCCATCAAGGTTCC |
| *PdbNF-YC7* | AATGCCTGCTGCCTCAAATC | TGTGATTCCAAGATGGTGCCT |
| *PdbNF-YC8* | TGGAACAAGTCCGTATCCGC | GATCCTAGCCAACGGAAGGC |
| *PdbMYC* | TTTGGGTCAGATCTTAGCGCTTATT | CAAATAAGACAGGCCTGGTTTATT |
| *PdbCOI* | ACCTTGAGCTTCTTGCCAGGAAC | TCTCCATGTAGCTAAGACCCAAAC |
| *PdbORCA* | AGAGGAGTGAGGCAAAGACCCT | TCCGGCTCTCGACGCTTAGT |
| *PdbJAZ* | CCTGCTTTGCCTGTCTATTTCCCTCT | GTTGGTGATCTGTTCTTGGATCGGGT |
| *PdbNPR 1* | GGCCGACGATACTTCCCTAGTT | TGCCCTCATAGTTTCCTGAGCT |
| *PdbPR1* | ACACCACCGTGCAAGCCTATG | CGAGCAGAGTTACGCCAAACCA |
| *PdbTGA* | GTAGCAGAGACTGTGGCAGC | GCCGTTGTAAGTATTCTCCTAAGGC |
| *PdbEF* | TGGGTCGTGTTGAAACTGGTGT | GGCAGGATCGTCCTTGGAGTTC |
| *Pdbactin* | GCTGAGAGATTCCGTTGCCCTG | GGCGGTGATCTCCTTGCTCATT |

Primers used in constructing plant plasmids

| Genes | Forward and reverse primers(5’-3’) | |
| --- | --- | --- |
| pROKII-PdbNF-YA11 | ATGCGGATCCATGGCTTTACGAATTC | GCATGAGCTCTCACTGAACAACGGAA |
| pFGC5941-PdbNF-YA11 | PdbNF-YA11-Cis-F: | PdbNF-YA11-Cis-R: |
|  | ATCGCCATGGGTTAGTTGCCCATCATGGTG | ATGCGACGTCAGATGAAATACATTGATCTTGTGAA |
|  | PdbNF-YA11-Anti-F: | PdbNF-YA11-Anti-R: |
|  | ATCGTCTAGAGTTAGTTGCCCATCATGGTG | ATGCGGATCCAGATGAAATACATTGATCTTGTGAA |
